# Intraspecific demographic and trait responses to environmental change drivers are linked in two species of ciliate

**DOI:** 10.1186/s12862-024-02241-2

**Published:** 2024-04-17

**Authors:** Tessa de Bruin, Frederik De Laender, Julie Jadoul, Nicolas Schtickzelle

**Affiliations:** 1 Earth and Life Institute (ELI), Biodiversity Research Center (BDIV), Université Catholique de Louvain, Louvain‑La‑Neuve, Belgium; 2 Research Unit in Environmental and Evolutionary Biology (URBE), Institute of Life-Earth-Environment (ILEE), Namur Institute for Complex Systems (NAXYS), Université de Namur, Namur, Belgium

**Keywords:** Intraspecific trait variation, Environmental change drivers, Population dynamics, Ciliates, Protists, Experimental microcosms

## Abstract

**Background:**

Over the past decade, theory and observations have suggested intraspecific variation, trait-based differences within species, as a buffer against biodiversity loss from multiple environmental changes. This buffering effect can only occur when different populations of the same species respond differently to environmental change. More specifically, variation of demographic responses fosters buffering of demography, while variation of trait responses fosters buffering of functioning. Understanding how both responses are related is important for predicting biodiversity loss and its consequences. In this study, we aimed to empirically assess whether population-level trait responses to multiple environmental change drivers are related to the demographic response to these drivers. To this end, we measured demographic and trait responses in microcosm experiments with two species of ciliated protists. For three clonal strains of each species, we measured responses to two environmental change drivers (climate change and pollution) and their combination. We also examined if relationships between demographic and trait responses existed across treatments and strains.

**Results:**

We found different demographic responses across strains of the same species but hardly any interactive effects between the two environmental change drivers. Also, trait responses (summarized in a survival strategy index) varied among strains within a species, again with no driver interactions. Demographic and trait responses were related across all strains of both species tested in this study: Increasing intrinsic growth and self-limitation were associated with a shift in survival strategy from sit-and-wait towards flee.

**Conclusions:**

Our results support the existence of a link between a population’s demographic and trait responses to environmental change drivers in two species of ciliate. Future work could dive deeper into the specifics of phenotypical trait values, and changes therein, related to specific life strategies in different species of ciliate and other zooplankton grazers.

**Supplementary Information:**

The online version contains supplementary material available at 10.1186/s12862-024-02241-2.

## Background

According to the global assessment report on biodiversity and ecosystem services [1], the main direct anthropogenic threat to biodiversity is the enhancement of co-occurring environmental change drivers, such as land/sea-use change, resource extraction, pollution, invasive/alien species, or climate change. For example, increased land use change leads to fragmentation of non-urban habitats, which in turn tends to increase temperature and air pollution [1–4]. Whether the total effect on biodiversity of two or more interacting drivers is antagonistic, synergistic or additive is difficult to predict [5–8]. This is because interactions within and between species in an ecosystem are, in both direct and indirect ways, affected by environmental change. The more drivers, the more complex it is to accurately predict community responses [7, 9].

The biodiversity decline caused by anthropogenically enhanced environmental change drivers has major destabilizing effects on ecosystem functions, like biomass production and nutrient uptake efficiency, which in turn affect ecosystem services essential for our society like food production, water supply and waste decomposition [10–13]. While classically, biodiversity has often been based on taxonomy, it has become increasingly apparent that trait-based diversity is a better predictor of ecosystem functioning as it results in a more nuanced view of species’ functioning and interactions within an ecosystem [13, 14]. Trait-based diversity occurs at multiple scales, from within-individual variation (phenotypic plasticity) to variation between functional groups [15], but is most often quantified at the species level (interspecific trait variation) [16–19]. Interspecific trait variation has been linked to demography, community composition and ecosystem functioning [20–24]. For example, Leary & Petchey (2009) showed that simple communities comprised of two species of protist were more stable (biomass fluctuated less) with increasing variation between the two species regarding demographic response (measured as intrinsic growth rate and carrying capacity) to temperature fluctuations [24]. Over the past decade, theory and observations suggest that variation of traits within species (intraspecific trait variation i.e. ITV) can be equally important as interspecific trait variation: ITV can affect a species’ demography, which in turn affects coexistence and interactions with other species present, influencing community-level biodiversity and increasing the stability of ecosystems subjected to anthropogenically induced environmental change drivers [16, 17, 25–31].

High interspecific diversity can create functional redundancy among species, making the ecosystem more resistant to environmental change drivers [32]. Similarly, ITV can theoretically create intraspecific functional redundancy among groups of phenotypically different individuals, which might respond differently to the same driver. If so, a population with a higher ITV is expected to be more resistant to environmental change drivers [33, 34]. However, the number of studies addressing whether ITV can indeed influence a population’s demographic response to anthropogenically induced environmental change driver effects is scarce, especially when considering experimental studies testing multiple, possibly interacting, environmental change drivers [35]. Despite results from theoretical studies emphasizing the importance of ITV for population and, ultimately, ecosystem dynamics [18, 36], our understanding of the modulating role of ITV in environmental change effects on population dynamics is limited [35, 37]. One of the current challenges is to track intraspecific trait change over time, as traits can be plastic and change in response to the environment [38, 39]. Moreover, the importance of considering multiple traits contributing to the same trait type (e.g. trait syndromes) is still often overlooked ([35], but see [40, 41]). Finally, when studying demography, it is important to include density dependence as this is a key aspect of population growth and is known to be affected by environmental change drivers [42–44].

In this study, we aim to empirically assess whether population-level responses in survival strategy to multiple environmental change drivers can explain the population-level demographic response to these same drivers. We expect that 1) in terms of demography, different populations of the same species respond differently to environmental change drivers, 2) individuals within a population respond to environmental change drivers by changing relevant phenotypical trait values, and 3) the population-level demographic response to environmental change drivers is related to changes in survival strategy. To test this, we performed experiments in controlled aquatic microcosms, using image analysis to characterize population demography and individual phenotypical traits of three clonal strains of two species of ciliated protists (*Colpidium striatum* and *Tetrahymena thermophila*) exposed to two environmental change drivers (climate change and pollution) and their combination. We first quantified density dependent demography, hereafter referred to as population demography, by measuring the intrinsic growth rate µ and the interaction coefficient between conspecifics α (combined in carrying capacity K). We measured how demography responded to environmental change for different strains of the same species. We then tested if these responses (due to environmental change drivers) were related to changes in mean and variance of a survival strategy comprised of individual movement and morphological traits.

## Methods

### Model species

*Tetrahymena thermophila* and *Colpidium striatum* are both bactivorous ciliates naturally occurring in temperate lakes, rivers, ponds and the like [45, 46].They are excellent model systems, since they are easy to maintain in the lab and grow relatively fast. When grown according to our lab setup (see “Culture conditions” section), *T*. *thermophila* reaches carrying capacity in about five, and *C*. *striatum* in about seven days. A broad selection of *T*. *thermophila* clonal strains was already well established in our lab at UCLouvain (see supplementary material of Pennekamp, 2014 [47] for details). For *C*. *striatum*, genetically diverse cultures were provided as a courtesy by the team of prof. O. Petchey, University of Zürich, Switzerland. Clonal strains were then created by isolating individual cells and allowing each of them to grow into a clonal population.

### Culture conditions

All ciliate stock cultures were kept in an incubator with a 14:10 h light:dark cycle at 20 °C in 250 mL square bottles with vented caps (DURAN®) containing 100 mL nutrient medium. The medium consisted of Chalkey’s solution supplemented with alfalfa powder (Allcura; 0.55 g/L) and bacterized with *Serratia fonticola*. Following bacterization, the medium was incubated for two days at 20 °C on a shaker to allow the bacteria to grow before ciliates were added. Cultures of *T*. *thermophila* were restocked every other week while those of *C*. *striatum*, growing more slowly, were restocked every third week.

In preparation for experiments, the nutrient medium (before bacterization) was filtered through a 0.22 µm filter system (BT50 500 mL) to remove all particulate matter. This filtered medium was then bacterized as described above and used for precultures and experimental microcosms. It was essential to use filtered medium for the experiments because the particulate matter would otherwise greatly impede the image analysis we use (see “Image analysis”).

### Experimental design

**Fig. 1:**
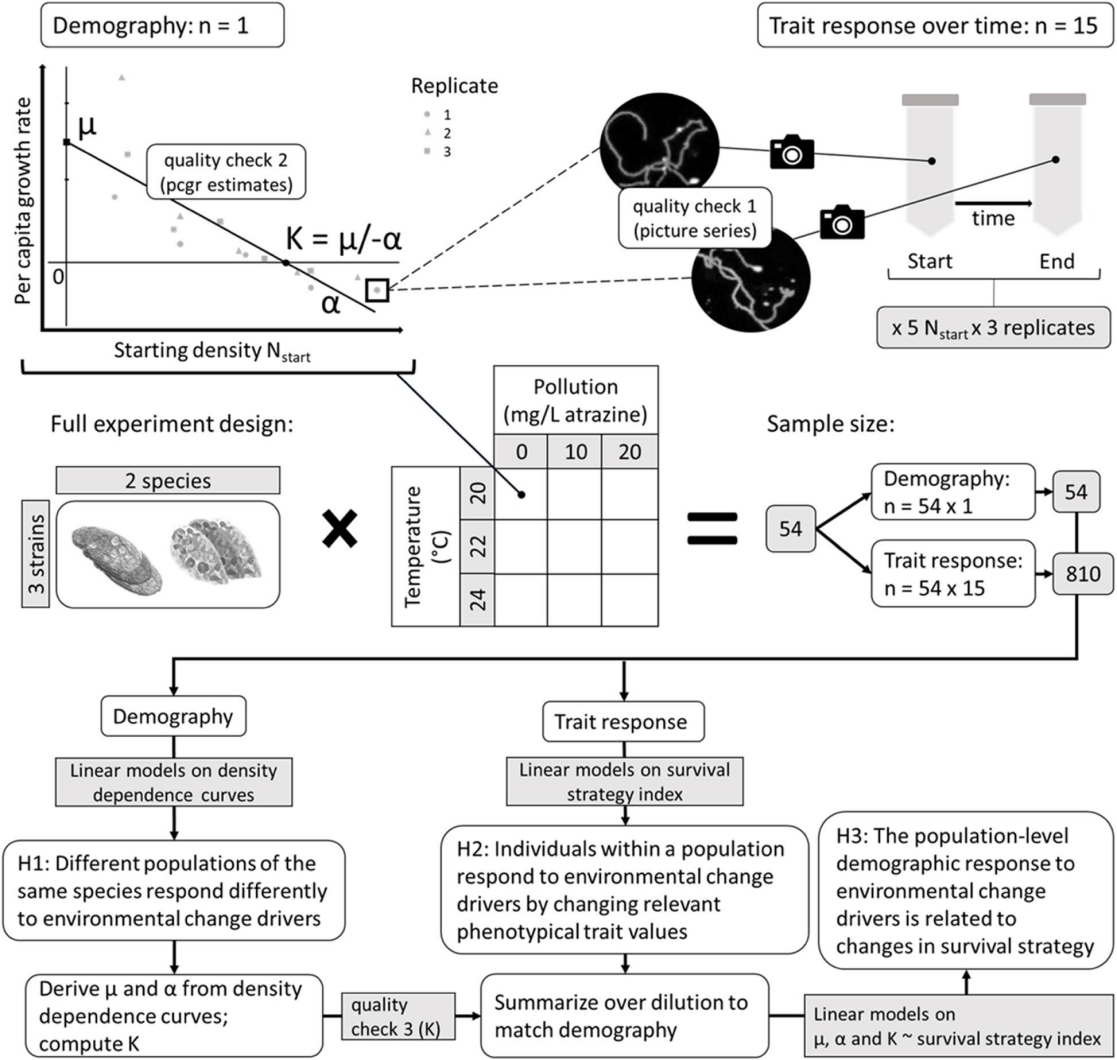
Visualized experimental design & analysis. Each density dependence curve originates from a dilution assay with 5 starting densities replicated thrice. Each curve therefore comprises of 15 datapoints that contribute to one set of demographic parameters and 15 sets of parameters describing response in movement and morphological traits. Each datapoint is constructed through image analysis of picture series from two sample points in time, T_start_ - T_end_. The full experimental design of temperature x pollution x species x strains yields a total of 54 density dependence curves, thus totaling to 54 parameter sets describing demography and 810 sets describing response in movement and morphological traits. Linear models on both datasets separately are used to test our first two hypotheses, after which linear models on the combined dataset are used to test our third hypothesis

The experiment involved three strains of each of the two ciliate species, each subjected to nine treatments consisting of three temperatures (20, 22, 24 °C) times three pollutant concentrations (0, 10, 20 µg/mL atrazine) in a fully crossed factorial design (Fig. 1). Atrazine, an herbicide, was chosen as a common pollutant in the framework of a larger multispecies project. Although ciliates are a non-target group, previous studies have shown atrazine to be toxic to ciliates as well [48, 49]. We verified the effect of atrazine on population growth of our ciliate species by means of a pilot experiment (Figure S1 in Additional file 2). For each of the 54 combinations (2 species * 3 strains * 3 temperatures * 3 pollutant concentrations), we performed three independent dilution assays, giving a total of 162 assays.

### Dilution assays

We quantified the density-dependence of the per-capita population growth rate (pcgr) by performing dilution assays. A dilution assay aims to measure population growth over a relatively short time interval according to the initial individual density in such a way that the only factor limiting growth is the resource availability per individual [38, 50]. For each strain of each species, a source culture was prepared to produce a population in its exponential growth (log-phase) at the start of the assay (i.e. 5 and 7 days in advance for *T*. *thermophila* and *C*. *striatum*, respectively). A series of microcosms corresponding to 5 different initial densities was then prepared using 20, 35, 50, 65 and 80% source culture in bacterized filtered medium with a total volume of 10 mL in 50 mL tubes. The appropriate amount of atrazine was added dissolved in 10 µL dimethylsulfoxide (DMSO). Microcosms subjected to 0 mg/L atrazine received 10 µL DMSO without added atrazine. Consecutively, 1 mL samples were taken from each microcosm to measure initial (${{\text{t}}}_{0}$) density, morphology and movement traits; the microcosms were then incubated at the appropriate temperatures and samples were again taken at two other times (${{\text{t}}}_{1}$ and ${{\text{t}}}_{2}$). The reason for using two additional times instead of one was that in some cases growth over time interval ${{\text{t}}}_{0}\to {{\text{t}}}_{1}$ was too low to be precisely measured; growth over ${{\text{t}}}_{0}\to {{\text{t}}}_{2}$ or ${{\text{t}}}_{1}\to {{\text{t}}}_{2}$ was used for these microcosms instead (Table S1 in Additional file 1). Under these assay conditions, population growth is expected to decrease exponentially with density so pcgr can be regressed against starting density to estimate two basic demographic parameters (Fig. 1) [38]: the intrinsic growth rate (µ) and the interaction coefficient between conspecifics (α):

1 $$\frac{{\text{ln}}\left({{\text{N}}}_{{\text{end}}}/{{\text{N}}}_{{\text{start}}}\right)}{{{\text{t}}}_{{\text{end}}}-{{\text{t}}}_{{\text{start}}}}=\mu +\alpha {{\text{N}}}_{{\text{start}}}$$

where ${{\text{N}}}_{{\text{start}}}$ and ${{\text{N}}}_{{\text{end}}}$ are the densities at the start and at the end of the appropriate time interval ${{\text{t}}}_{{\text{start}}}\to {{\text{t}}}_{{\text{end}}}$ (Table S1 in Additional file 1). Carrying capacity (K) was then defined as -µ/α. The variance of K was computed using a Taylor approximation according to mean, variance and covariance of µ and α [51]:

2 $${s}^{2}\left(\frac{-\mu }{\alpha }\right)\approx \frac{{\left({\overline{x} }_{\mu }\right)}^{2}}{{\left({\overline{x} }_{\alpha }\right)}^{2}}\left(\frac{{s}_{\mu }^{2}}{{\left({\overline{x} }_{\mu }\right)}^{2}}-2\frac{\mathit{cov}\left(\mu ,\alpha \right)}{{\overline{x} }_{\mu }{\overline{x} }_{\alpha }}+\frac{{s}_{\alpha }^{2}}{{\left({\overline{x} }_{\alpha }\right)}^{2}}\right)$$

For each of the 54 experimental conditions, we chose to fit one single density dependence curve over the three replicate dilution assays. Such an approach has been proven to maximize the precision of parameter estimates in such a regression design where X (here ${{\text{N}}}_{{\text{start}}}$) is a continuous predictor [52]. In total, each regression curve contained 15 data points (5 dilutions * 3 replicates) and produced one estimate of µ and of α.

### Image analysis

To measure population demographic as well as individual morphological and movement traits, we used an updated version of the method described by Pennekamp, Schtickzelle & Petchey [53, 54]. A custom-made counting slide and imaging platform at our lab allowed for us to take pictures of 20 samples sequentially, greatly reducing sampling time and optimizing work flow. In short, for each sample, a series of pictures was taken of 810 µL using a Sony A9 camera equipped with an FE 90 mm F2.8 Macro G OSS lens and a darkfield approach (indirect light and black background). Each series consisted of 10 s burst shots at 10 fps in grey scale (other settings: 1/160 s, F11, ISO 6400), resulting in 100 pictures per sample.

Automated image analysis using Fiji [55] incorporated in a Python 3 script [56] was used to identify, track and characterize all moving particles (i.e. individual ciliates) in the sample [53, 54]. This analysis involved three steps: first (particle analysis), each image was assessed separately to identify and characterize all particles considered as ciliates, i.e. fitting the constrains of a set of parameters describing grey scale values (to discriminate between white particles and black background), cell size (surface area in pixels) and cell shape (aspect ratio, i.e. major/minor axis of a fitted ellipse). Second (particle tracking), ciliates identified on consecutive pictures were compared and associated according to constrains describing possible movement speed and distance, allowing to discriminate ciliates from e.g. moving artefacts, leading to the reconstruction of trajectories of individual ciliate cells. Movement speed was computed as gross displacement over displacement duration and linearity as gross over net displacement of a given ciliate individual, net displacement being the straight-line distance from start to end position. Third (movement analysis), executed in R (v4.3.0; using the circular, dplyr and data.table packages, [57–59]), the particle tracking data were used to calculate the mean traits in terms of movement and morphology of each trajectory within a picture series.

After movement analysis, any trajectory not fitting minimal quality parameters (in terms of duration, displacement and cell detection frequency) was discarded. All parameter values were manually finetuned per species to optimize ciliate detection and minimize the chance of artefacts being mislabeled as ciliates (see Tables S2 and S3 in Additional file 1 for a detailed overview of all parameter constrains). Density was defined as the number of trajectories detected in a sample corrected for sample volume. To ensure equal sample size and analysis at the appropriate replication level (the sample), phenotypical traits were averaged over all trajectories in a given sample (population level). The resulting dataset therefore comprised of one mean and one variance value per trait of interest for each of the 2430 pictures series (i.e. 162 assays * 5 starting densities * 3 time points): ciliate morphology (cell size and shape) and movement (speed and linearity).

### Data quality check

Despite the efficiency of our experimental protocols and video analysis procedure, we applied data quality checks at several steps of the analysis.

First, after image analysis, boxplots were plotted for each trait per strain and picture series of samples marked as outliers were visually inspected. If pictures were not up to standard (e.g. the focus was off, biasing morphology measurements, or too many artefacts were tracked, biasing density, morphology and movement measurements), the sample was discarded. When the focus was off, sometimes cells were tracked correctly if we reran image analysis for that specific sample with adjusted tracking parameters. In those cases, we could still use the density measurement. From the total of 2430 samples, *n* = 2408 were up to quality standard regarding density and *n* = 2382 remained for which picture series were up to quality standard regarding movement and morphology traits.

Second, density measurements resulting from the image analysis (*n* = 2408, after the first quality check) were used to construct the density dependence curves. The best time interval was chosen for each of three replicate assays separately, and the resulting per capita growth rate (pcgr) estimates contributed to one single density dependence curve (see “Dilution assays” section; Fig. 1). Note that from here on, only data from time points contributing to the density dependence curves was used in further analysis. From the original 810 sample pairs, *n* = 741 remained for which data on both correct time points was complete regarding trait (movement & morphology) measurements, while *n* = 807 were complete regarding density measurements. This resulted in 807 measured pcgr estimates, contributing to 54 density dependence curves (Fig. 1; Figure S2 in Additional file 2). Equation 1 was fitted to each curve, and model diagnostics plots were used to assess the quality of the pcgr estimates. We checked for residual nonlinearity and found none, indicating that a linear model was indeed the best approach for fitting the data. However, we had reason to believe that some pcgr estimates were affected by a ‘failed’ assay (e.g. very little growth occurred or the culture even collapsed) or a sampling error at one of the time points (cells cluster together sometimes despite carefully mixing the culture before sampling). Therefore, we decided to exclude a point if 1) it was outside or close to the border of Cook’s distance AND 2) marked as an influential point in the other three (residuals vs fitted, qq and scale location) plots. This way, an additional 40 points were excluded from contributing to the density dependence curves, but not from contributing to the trait (movement & morphology) data. We supplied density dependence curves with and without these 40 points in our supplementary material (Figure S2 in Additional file 2).

Third, the estimated values and variances of µ and α obtained from each of the 54 regressions were combined to compute K estimates as per Eq. 2 (Fig. 1). We discarded 3 estimates of K that were extremely negative (K < -400) because such values signified positive density dependence (e.g. α was positive meaning per capita growth rates increased with increasing density). In those cases, K estimated from the density dependence curve was not a meaningful demographic parameter, as growth was not limited by density.

### Statistical analysis

All statistical analysis was performed using R Statistical Software (v4.3.0) [60].

#### Demography

To assess whether µ and α differed per strain and/or treatment within a species, a linear model assessing what factors affected the density dependence curves (i.e. pcgr ~ ${{\text{N}}}_{{\text{start}}}$) was performed for each species separately. To test for differences in µ (i.e. y-intercept of the density dependence curve), we tested pcgr against all factors separately as well as up to 3-way interactions between strain and/or temperature and/or atrazine pollution while, to test for differences in α (i.e. slope of the density dependence curve), we needed to test for up to four-way interactions between ${{\text{N}}}_{{\text{start}}}$ and all other factors.

#### Movement & morphology

All movement and morphology traits were standardized at the species level and combined in an index using the formula: index = speed + linearity—size + shape. This index expresses the population positioning in terms of survival strategy from a sit-and-wait strategy (small index value) to a flee strategy (high index value). Cells trying to leave the environment were expected to move fast (high speed) and in a straight line (high linearity) while being relatively small (low size) and elongated (high shape). Opposite index values characterized cells staying in the environment. This specific combo was chosen based on the following assumptions: 1) moving fast in a straight line is the most energy efficient way of dispersal [61]. 2) The most energy efficient shape to move through a viscous environment is an elongated sphere (= high aspect ratio) [62] and 3) no or little energy would be available for cell growth, hence the high aspect ratio and small size. Since we were interested in the relation between trait response and demography, we computed the response in mean and variance of the index (Δmean index and Δvariance index, respectively) over the same time interval as used to compute growth. We then tested the effects of and interactions between strain, temperature and atrazine pollution on Δmean index and Δvariance index for each species separately using a linear model.

#### Population demography explained by survival strategy response

Prior to this step, all demographic parameters (µ, α and K) were standardized at the species level. In order to compare the demographic versus the index response values, we summarized the latter over replicates and dilutions to obtain one set of trait-response values for each set of demographic parameters (*n* = 54, see Fig. 1). To test whether the observed differences in intraspecific demographic trait values (µ and α separately, as well as summarized by K) can be explained by changes in mean and/or variance in survival strategy, we fitted several variants of a linear model of each demographic trait (µ, α or K) according to Δmean and Δvariance of the index, where the intercept and/or the slope was allowed to differ among species or (sets of) strains, using one datapoint per dilution assay (so Δmean or Δvariance index averaged over replicate and dilution to go with one value of µ, α or K). We used AICc model selection [63] to select the model variant best describing the data.

## Results

### Demography

**Fig. 2:**
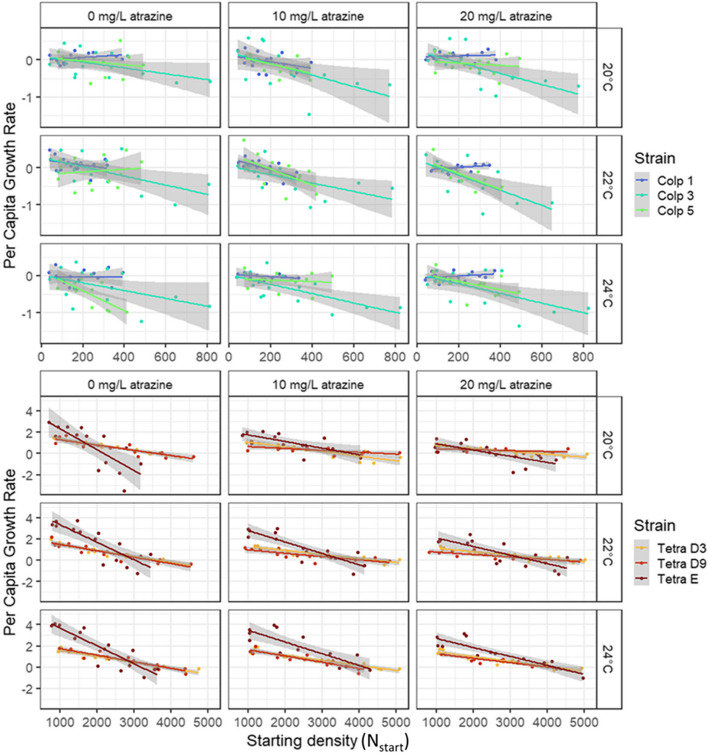
Density dependence curves per strain and treatment ± 95% CI for *C. striatum* (Colp) and *T. thermophila* (Tetra)

**Fig. 3:**
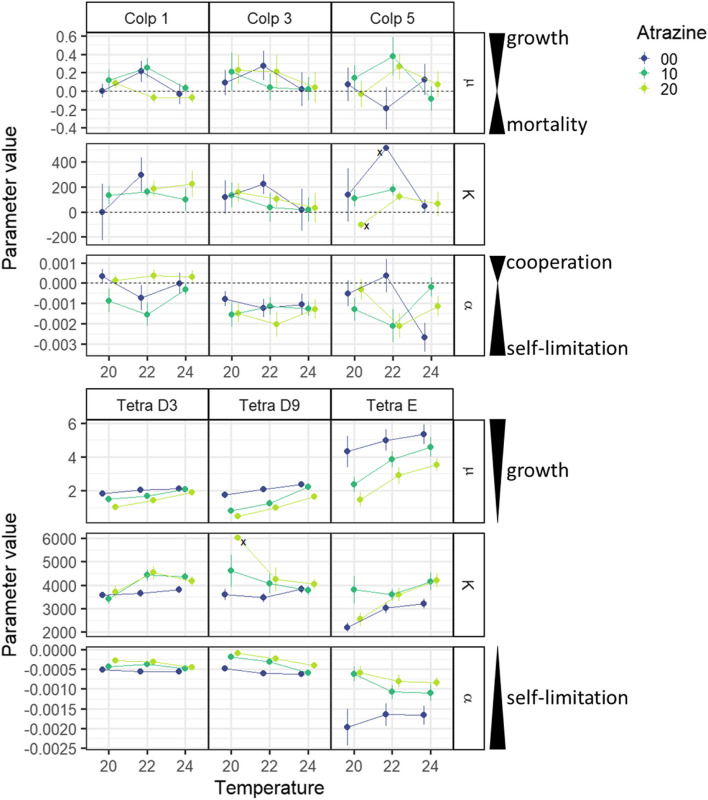
Mean ± SD of demographic parameters for *C. striatum* and *T. thermophila* per strain, temperature and atrazine. Some values of K are marked with an ‘x’; these values are displayed without their SD values because those were so large ( $\gg \overline{{\text{s}} }+2{\text{SD}}\left({\text{s}}\right)$ where $\overline{{\text{s}} }$ was calculated at species level regardless of treatment) that they greatly impeded the readability of the figure. These marked values of K are thus less reliable than the ones with lower SD values

Our analysis of the density dependence curves (Table 1, Figs. 2 and 3) confirms that both the intrinsic growth rate µ and the interaction coefficient α differed among strains, illustrating the presence of ITV in demography concerning these two species of ciliate. In *C. striatum*, intrinsic growth rate µ differed among strains and was affected by temperature in a nonlinear way. The temperature effect on µ differed somewhat depending on the atrazine pollution treatment, but this interaction effect was rather small and inconsistent among strains (pairwise comparisons using lsmeans package; Table 1, Fig. 3) [64]. Neither temperature nor atrazine had an effect on interaction coefficient α, but α differed among strains. Finally, looking at the effect sizes of the model factors expressed as partial eta squared (${\eta }_{p}^{2}$; [65]) all effects were quite small except the general slope (α). In other words, ITV in demography concerning *C. striatum* strains used in this study was mostly due to differences among strains regarding interactions between individuals. In *T. thermophila*, both µ and α differed among strains and were impacted by temperature and atrazine without any interaction effects. Intrinsic growth rate µ increased with temperature for all strains, with the strength of the effect differing among strains, while interaction coefficient α decreased only for strain Tetra D9 (i.e. self-limitation increased; Table 1, Fig. 3). For atrazine, µ decreased with atrazine pollution only for strain Tetra E, while α increased for Tetra D9 and Tetra E. The strength of the effect differed among strains regarding α. Finally, the effect sizes were notably larger for *T. thermophila* than for *C. striatum*. In other words, ITV in demography concerning *T. thermophila* strains used in this study was quite strong and due to differences among strains regarding their base demography and regarding their demographic response to environmental change drivers of which temperature had a stronger effect than atrazine pollution.

**Table 1:** Output of the linear model testing the effects of strain, temperature, atrazine and their interactions on intrinsic growth rate µ and interaction coefficient α, for *C. striatum* and *T. thermophila*. Significant *p*-values are in bold and accompanied by the effect size of the model factor, expressed as partial eta squared. The main model factors express differences in µ (intercept) while interaction of these factors with N_start_ express differences in α (slope)

**C. striatum**		**µ (main factor)**			**α (interaction with ** ${\mathbf{N}}_{\mathbf{s}\mathbf{t}\mathbf{a}\mathbf{r}\mathbf{t}}$**)**		
**model factors (pcgr ~ …)**	**df**	***F*-value**	***p*-value**	${{\varvec{\upeta}}}_{\mathbf{p}}^{2}$	***F*-value**	***p*-value**	${{\varvec{\upeta}}}_{\mathbf{p}}^{2}$
Starting density (${{\text{N}}}_{{\text{start}}}$)	1	-	-	-	144.213	**< 2e-16**	**0.30**
Atrazine	2	0.970	0.380		2.064	0.129	
Temperature	2	6.099	**0.003**	**0.04**	0.452	0.637	
Strain	2	8.086	**0.000**	**0.05**	6.656	**0.001**	**0.04**
Atrazine * Temperature	4	2.799	**0.026**	**0.03**	1.046	0.384	
Atrazine * Strain	4	1.295	0.272		0.916	0.454	
Temperature * Strain	4	0.332	0.857		0.242	0.915	
Atrazine * Temperature * Strain	8	0.702	0.690		1.838	0.069	
Residuals	330						
**T. thermophila**		**µ (main factor)**			**α (interaction with ** ${\mathbf{N}}_{\mathbf{s}\mathbf{t}\mathbf{a}\mathbf{r}\mathbf{t}}$**)**		
**model factors (pcgr ~ …)**	**df**	***F*-value**	***p*-value**	${{\varvec{\upeta}}}_{\mathbf{p}}^{2}$	***F*-value**	***p*-value**	${{\varvec{\upeta}}}_{\mathbf{p}}^{2}$
Starting density (${{\text{N}}}_{{\text{start}}}$)	1	-	-	-	652.510	**< 2e-16**	**0.66**
Atrazine	2	6.211	**0.002**	**0.04**	25.389	**0.000**	**0.13**
Temperature	2	42.767	**< 2e-16**	**0.21**	8.985	**0.000**	**0.05**
Strain	2	24.608	**0.000**	**0.13**	67.994	**< 2e-16**	**0.29**
Atrazine * Temperature	4	0.257	0.906		0.685	0.603	
Atrazine * Strain	4	3.962	**0.004**	**0.05**	7.358	**0.000**	**0.08**
Temperature * Strain	4	12.714	**0.000**	**0.13**	1.035	0.389	
Atrazine * Temperature * Strain	8	1.056	0.394		0.783	0.618	
Residuals	329

### Movement & morphology

**Fig. 4:**
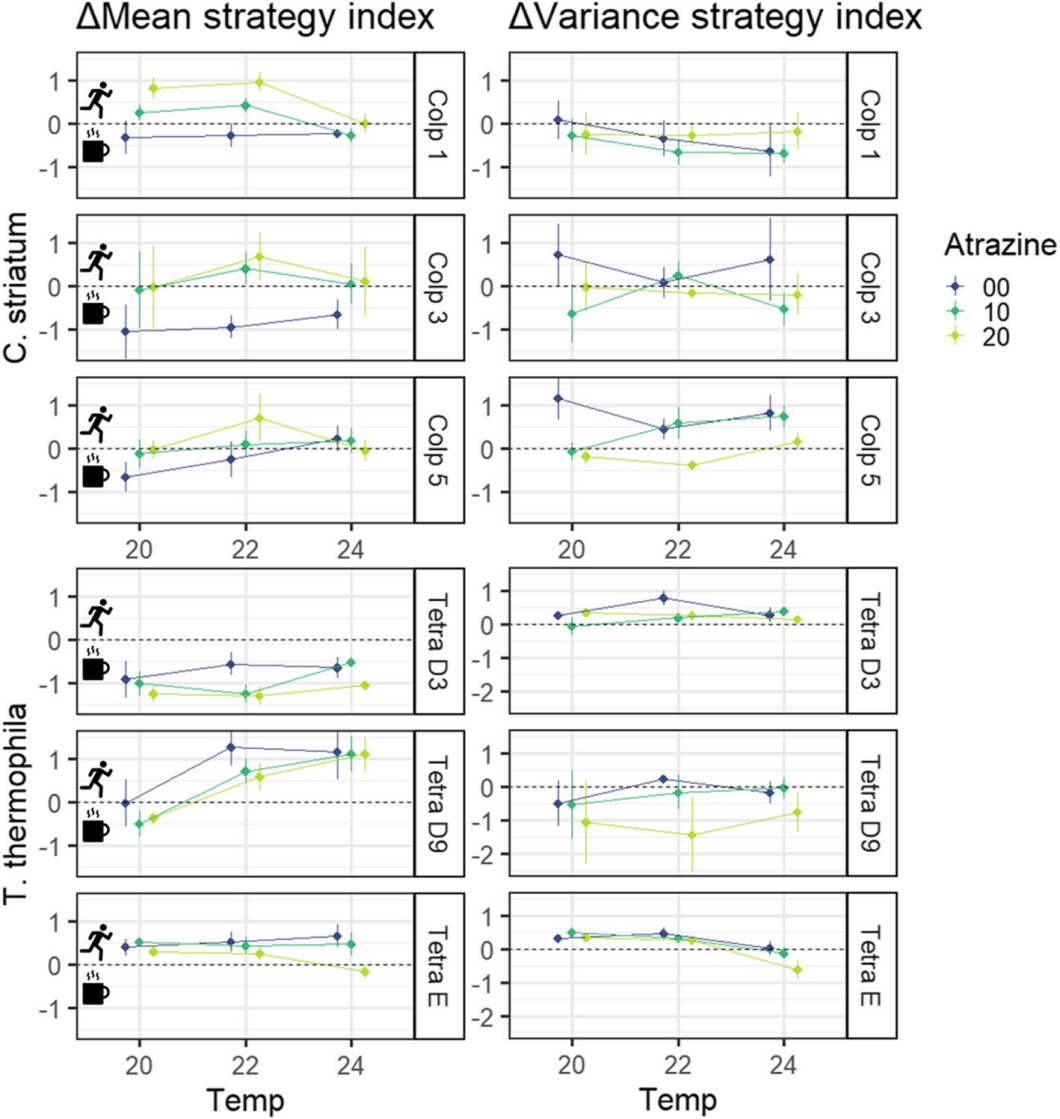
Change in strategy index per strain, temperature and atrazine concentration. Points are the response in mean and variance of the survival strategy index (ΔMean and ΔVariance, respectively). A positive ΔMean index suggests ciliates changed their trait values in accordance with increased dispersal behavior and vice versa for a negative ΔMean index

The trait-specific results per each time point as well as the response per time interval are available in our supplementary material (Figures S3-S6 in Additional file 2). Our analysis of the survival strategy index response (Table 2, Fig. 4) found differences due to species, strains and separate environmental change drivers. However, environmental change driver effects did not interact for any of the strains tested. Regarding *C. striatum*, atrazine pollution had an increasing effect on the mean index response (Δmean index) that was similar for all three strains while no effects of temperature or strain were found (Fig. 4; Table 2); this indicates that with increasing atrazine concentration, cells increasingly changed their strategy from sit-and-wait towards flee over the course of the time interval measured. The variance of the index response (Δvariance index) differed among strains and atrazine pollution had an overall decreasing effect, meaning that *C. striatum* strains used in this study varied in their plasticity regarding strategy index response and that with increasing atrazine concentration, this plasticity decreased. All effect sizes were medium (${\eta }_{p}^{2}$ ≥ 0.06) and the total variance explained was low for both models (adjusted *R*^2^ = 0.05 and 0.06 for Δmean and Δvariance, respectively), suggesting that survival strategy was not too greatly impacted by our treatments, or at least not in a linear way. For *T. thermophila*, strains strongly differed in their mean strategy index response, with Tetra D3 shifting to a sit-and-wait strategy and Tetra E towards a flee strategy whatever the temperature or atrazine concentration, while Tetra D9 showed a shift towards a flee strategy but only above 20 °C. Furthermore, increasing temperature was associated to a shift towards a flee strategy, much more pronounced for Tetra D9 than for the other two strains. Atrazine was associated to a shift towards the sit-and-wait strategy, similar in strength for all strains of *T. thermophila*. Therefore, strains of *T. thermophila* responded in the opposite direction in terms of survival strategy than strains of *C. striatum* when exposed to atrazine pollution. Regarding the variance of the index response, no effects were found except that Δvariance differed among strains, meaning that *T. thermophila* strains used in this study varied in their plasticity regarding strategy index response (Fig. 4; Table 2). However, post-hoc tests suggested the difference in Δvariance index among strains was mostly due to Tetra D9 being negatively affected by the highest atrazine treatment (pairwise comparisons using lsmeans package; 64). All effect sizes were large (${\eta }_{p}^{2}$ ≥ 0.14) except for the main effects of atrazine and temperature regarding Δmean index, which were medium (${\eta }_{p}^{2}$ ≥ 0.06). Additionally, the total variance explained regarding the Δmean index model was quite high (adjusted *R*^2^ = 0.56 and 0.06 for Δmean and Δvariance, respectively), suggesting that survival strategy was quite strongly impacted by our treatments.

**Table 2:** Output of linear models testing the effects of and interactions between atrazine pollution (A), temperature (T) and strain on the response in mean and variance of the survival strategy index (Δmean index and Δvariance index, respectively). Significant *p*-values are in bold and accompanied by the effect size of the model factor, expressed as partial eta squared

**C. striatum model factors (Δmean index ~ …)**	**df**	***F*-value**	***p*-value**	${{\varvec{\upeta}}}_{\mathbf{p}}^{2}$	**C. striatum model factors (Δvariance index ~ …)**	**df**	***F*-value**	***p*-value**	${{\varvec{\upeta}}}_{\mathbf{p}}^{2}$
Atrazine	2	8.297	**0.000**	**0.13**	Atrazine	2	3.567	**0.032**	**0.06**
Temperature	2	1.508	0.226		Temperature	2	0.136	0.873	
Strain	2	1.259	0.288		Strain	2	5.966	**0.004**	**0.10**
Atrazine * Temperature	4	1.114	0.354		Atrazine * Temperature	4	0.951	0.438	
Atrazine * Strain	4	0.824	0.513		Atrazine * Strain	4	1.443	0.225	
Temperature * Strain	4	0.706	0.590		Temperature * Strain	4	0.458	0.766	
Atrazine * Temperature * Strain	8	0.089	0.999		Atrazine * Temperature * Strain	8	0.425	0.904	
Residuals	108				Residuals	103			
**T. thermophila model factors (Δmean index ~ …)**	**df**	***F*-value**	***p*-value**	${{\varvec{\upeta}}}_{\mathbf{p}}^{2}$	**T. thermophila model factors** **(Δvariance index ~ …)**	**df**	***F*-value**	***p*-value**	${{\varvec{\upeta}}}_{\mathbf{p}}^{2}$
Atrazine	2	4.356	**0.015**	**0.08**	Atrazine	2	2.294	0.106	
Temperature	2	8.347	**0.000**	**0.13**	Temperature	2	0.431	0.651	
Strain	2	68.293	**0.000**	**0.56**	Strain	2	8.206	**0.000**	**0.14**
Atrazine * Temperature	4	0.469	0.758		Atrazine * Temperature	4	0.540	0.707	
Atrazine * Strain	4	0.325	0.861		Atrazine * Strain	4	1.115	0.354	
Temperature * Strain	4	5.986	**0.000**	**0.18**	Temperature * Strain	4	0.960	0.433	
Atrazine * Temperature * Strain	8	0.391	0.923		Atrazine * Temperature * Strain	8	0.238	0.983	
Residuals	107				Residuals	92

### Population demography explained by survival strategy response

**Fig. 5:**
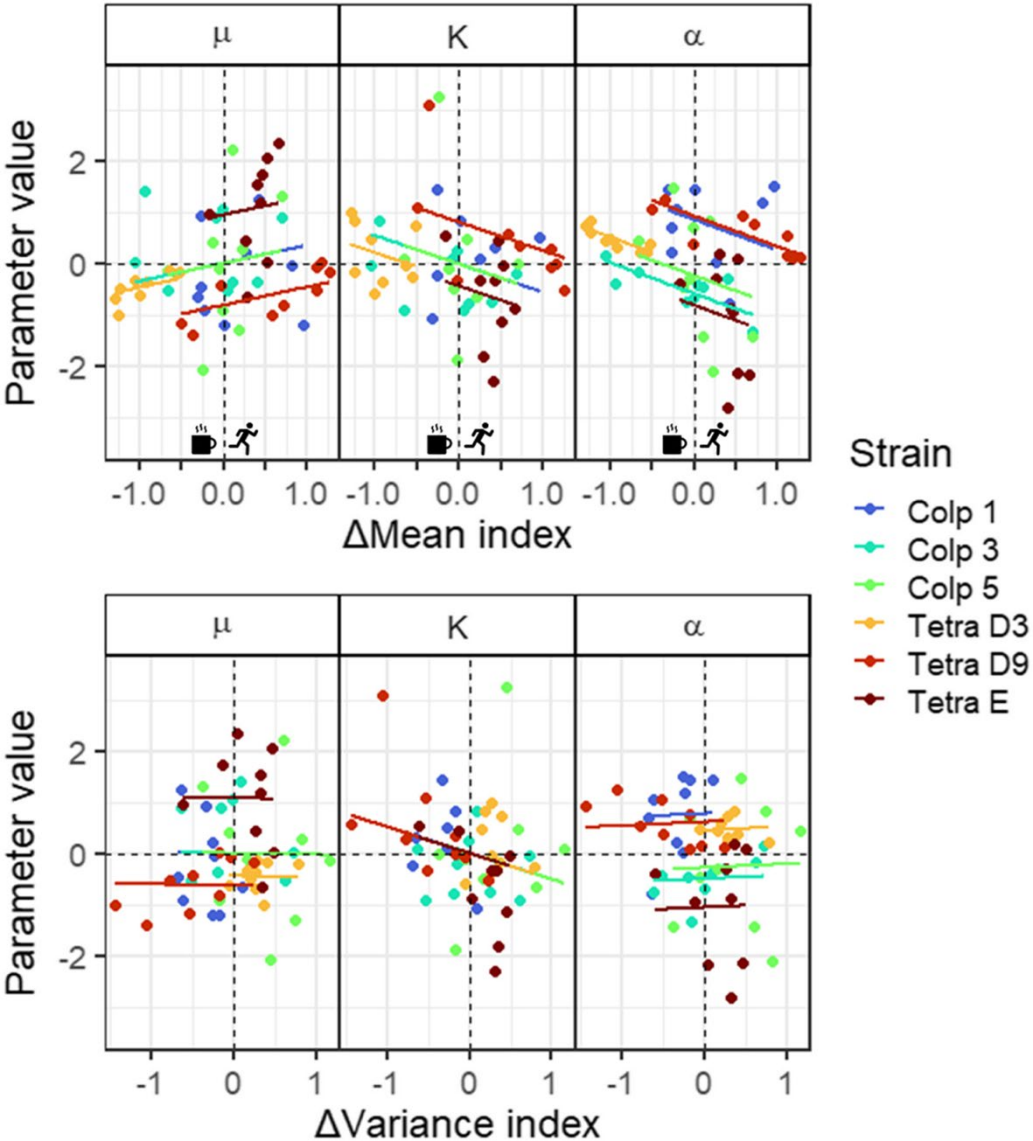
Demography (in the form of intrinsic growth rate µ, carrying capacity K and interaction coefficient α) explained by mean and variance of the survival strategy index response (Δmean and Δvariance index, respectively) according to the best model (see Table 3)

Our analysis of the survival strategy response explained by demography and strains, showed that each of the demographic parameters (intrinsic growth rate µ, interaction coefficient α and carrying capacity K) were related to the mean and/or variance of the survival strategy index (Table 3, Fig. 5). The best models describing the relation of µ and α to the mean index response (models Mean 4 and Mean 2, respectively; Table 3) showed a significant effect of Δmean. The direction of this effect was positive for µ and negative for α, and the intercept differed among strains of *T. themophila* only regarding µ but among all strains regarding α. This suggests that 1) ciliates shifted towards a flee strategy with increasing growth and self-limitation, 2) growth and self-limitation beyond a certain threshold value are associated with an increase in dispersal, and 3) the threshold value for growth differs more strongly intraspecifically for *T. thermophila* than for for *C. striatum*, while intraspecific variation regarding the threshold value for self-limitation is similar for both species. According to the best model describing the relation of K to Δmean (model Mean 4), there was no significant effect of Δmean, although AICc increased (e.g. the model fitted less well) if Δmean was left out of the model (data not shown). The slope of K ~ Δmean index in Fig. 5 is therefore not significantly different from zero, although there seems to be a negative trend similar to α ~ Δmean index. The intercept differed among strains of *T. themophila* only, suggesting that strains used in this study differ more strongly intraspecifically for *T. thermophila* than for *C. striatum* concerning their carrying capacity. Figures 3 and 4 support this: strains from *T. thermophila* differ more strongly in scale concerning both the demographic parameters and the Δmean index than strains of *C. striatum*.

**Table 3:** Comparison of linear models to test whether demography (in the form of intrinsic growth rate µ, carrying capacity K and interaction coefficient α) can be explained by the response in mean or variance of the strategy index (Δmean and Δvariance, respectively) and if this effect differs among and/or interacts with strains. StrainT indicates only strains of *T. thermophila* were allowed to have different intercepts while all *Colpidium* strains were set to have the same intercept and vice versa for StrainC. The best models are in bold

Model name	Model function (µ, K or α ~ …)	µ (*n* = 54)		K (*n* = 51)		α (*n* = 54)	
		**AICc**	**Adjusted R**^**2**^	**AICc**	**Adjusted R**^**2**^	**AICc**	**Adjusted R**^**2**^
Null	~ 1	155.44	-	146.94	-	155.44	-
Mean 1	~ Δmean	154.53	0.04	146.89	0.02	153.72	0.05
Mean 2	~ Δmean + strain	146.01	0.28	148.45	0.13	**131.74**	**0.45**
Mean 3	~ Δmean + strainC	159.77	0.02	152.46	0.00	149.15	0.19
Mean 4	~ Δmean + strainT	**143.57**	**0.27**	**145.66**	**0.12**	143.94	0.27
Mean 5	~ Δmean * strain	156.81	0.27	161.60	0.08	139.64	0.47
Mean 6	~ Δmean * strainC	165.49	0.00	159.95	-0.05	153.61	0.20
Mean 7	~ Δmean * strainT	149.56	0.26	151.45	0.11	150.23	0.25
Variance 1	~ Δvariance	157.05	-0.01	**145.65**	**0.05**	155.23	0.03
Variance 2	~ Δvariance + strain	149.12	0.24	151.94	0.07	**139.31**	**0.37**
Variance 3	~ Δvariance + strainC	163.26	-0.05	151.72	0.01	155.53	0.09
Variance 4	~ Δvariance + strainT	**145.68**	**0.24**	147.81	0.09	145.80	0.24
Variance 5	~ Δvariance * strain	161.06	0.21	160.83	0.09	149.54	0.36
Variance 6	~ Δvariance * strainC	167.91	-0.04	154.12	0.06	157.69	0.14
Variance 7	~ Δvariance * strainT	151.34	0.23	150.73	0.12	153.61	0.20

None of the best models describing the relation of µ, K and α to the variance of the index response (model Variance 4, Variance 1 and Variance 2, respectively) contained a significant relation between demography and Δvariance, which is apparent from Fig. 5. Furthermore, similar to model mean 2 and mean 4, the intercept differed among strains of *T. themophila* only regarding µ but among all strains regarding α. Since the slope did not differ from zero in both cases, this merely suggests that intraspecific variation in growth is stronger for *T. thermophila* than for for *C. striatum*, while intraspecific variation in self-limitation is similar for both species.

## Discussion

In this study, we aimed to empirically assess whether population-level responses in survival strategy to multiple environmental change drivers can explain the population-level demographic response to these same drivers. We used controlled aquatic microcosms and image analysis to characterize density dependent demography and functional traits of three clonal strains of two species of ciliated protists.

Firstly, we found that population demography responds differently to environmental change drivers for different strains of the same species but that there are barely any additional effects due to the two environmental change drivers interacting. Secondly, all clonal strain populations changed their survival strategy (mean and/or variance) in response to the environmental change drivers, but the nature of the response differed both between and within species. Thirdly, the response of demography to environmental change drivers in the form of intrinsic growth rate and self-limitation was linked to changes in survival strategy, but no such link was found when demography was described as carrying capacity. These results support the existence of a link between a population’s demographic and trait responses to environmental change drivers in two species of ciliate.

Temperature increase and pollution are two well-studied environmental change drivers known to interact [66–69]. The nature of their interaction is likely to depend on temperature-induced changes in metabolism, which can either enhance or mitigate pollution-related effects [69]. Since atrazine has been observed to reduce population growth in *Tetrahymena pyriformis* [48, 49], we expected to find some kind of interaction between the temperature and atrazine treatments. We found one such interaction in the demographic response to our environmental change driver treatments in *C. striatum*, but post-hoc tests suggested this was mainly due to the response of one strain (Colp 5). Therefore, temperature and atrazine effects in our experiment were additive except for the one case, which nicely illustrates the importance of intraspecific variation.

Regarding the response in mean strategy index, *T. thermophila* strains were much more different from each other than *C. striatum* strains. This can be explained by the fact that *T. thermophila* strains were grown from mono-strain axenic cultures of different spatial origin (isolated from several sites over a latitudinal gradient in North-America) that have never faced competition in the lab, while strains of *C. striatum* were created from a genetically diverse mother culture already maintained in the lab for some years. In other words, *C. striatum* was maintained in a mother culture for thousands of generations during which stabilizing evolution is expected to have limited its ITV. Additionally, while the response in mean index was for a substantial part (*R*^2^ = 0.56) explained by our treatments concerning *T. thermophila*, only a minor part (*R*^2^ = 0.05) was explained concerning *C. striatum*. There is thus likely some other factor that affects trait response that we did not test for.

The response of strain-specific variance regarding the strategy index was affected by the experimental treatments in a dissimilar way between species (Table 2, Fig. 4). Since each strain originated from one single cell and ciliates are known to be robust against mutations in the somatic nucleus (ciliates have two nuclei, but only the material in the somatic one is expressed during vegetative growth; [70, 71]), trait variance within strains in this study is most likely due to plasticity. Therefore, the treatment effects on the response of index variance suggest that 1) the plasticity in behavioral response differs among strains in both *C. striatum* and *T. thermophila* and 2) increasing atrazine pollution makes individual cells behave more similarly within strains of *C. striatum* only.

Our results show that population-specific density dependent demography in response to environmental change drivers is linked to the response of survival strategy to these same drivers. The best models explained 27% and 45% of variation in population-specific intrinsic growth (µ) and interaction coefficient (α), respectively, and suggested that ciliates shifted towards a flee strategy with increasing growth and self-limitation. When the demographic response was summarized as carrying capacity, however, we did not find such a link. This could suggest that using carrying capacity as a summarizing (derived) parameter for density dependent demography is too simplistic, emphasizing the importance to include density dependent growth and interactions when studying demography [42, 43].

Wieczynski et al. [38] already demonstrated the link between demography and several ciliate traits, among which size and shape. Our study provides additional data supporting their conclusions and adding movement and morphology to the list of phenotypical traits linked to demography. Moreover, through the use of a meaningful summarizing index, our study enables us to explain the link between demography and individual traits in a way that makes logical sense. Since we know from previous studies that traits are not independent from each other and “form” trait types, more specifically in this case behavioral syndromes [35, 40, 41, 72–75], we used a trait index based on the link between phenotypical trait values and ciliate behaviors previously observed in *T. thermophila* [72], rather than individual trait values, for the analysis. Importantly, we looked at the change in this index over the length of the experiment rather than just at the initial or final values, as traits can be plastic and change in response to the environment [38, 39]. We did so to get a more nuanced view of what happens survival strategy-wise in a population after a change in the environment.

Although the emphasis of this study is put on phenotypical traits explaining demography, we observed correlated variation in both, meaning the link between demographic and trait responses to environmental change drivers is reciprocal: The increase in survival strategy index for strain Tetra D9 in response to increasing temperature (Table 2; Fig. 4) could be due to an increased growth rate and, especially, self-limitation (Fig. 3). Denser populations do not necessarily stimulate dispersal as interactions among conspecifics can be beneficial, for example when secondary metabolites are shared between cells [76–78]. In addition to self-limitation, environmental factors and genetics play a non-negligible role in determining dispersal propensity [79–81]. In the case of the *T. thermophila* strains used in this study, Pennekamp et al. [72] found that Tetra E does, but Tetra D3 and D9 do not, show negative density-dependent dispersal. This means that, in the case of Tetra E, cells in denser populations tend to disperse less and vice versa. We found that, within *T. thermophila*, Tetra E was the most sensitive in its demographic response to the environmental change driver treatments and had overall high growth rate and strong self-limitation leading to relatively low carrying capacity (Fig. 3). Negative density dependence could explain why Tetra E decreased its mean survival index (i.e. population-specific mean trait values changed in such a way as to be less in accordance with dispersal behavior) in response to the 24 °C + 20 mg/L atrazine treatment only, as this treatment resulted in its highest K value. Concerning *C. striatum*, there is little information to be found about its density – dispersal relationship. Two studies tested for density dependent dispersal in microcosm experiments but the results were non-significant in both, suggesting density might not be a driving factor of dispersal in *C. striatum* [82, 83]. In our experiment, higher atrazine concentrations clearly increased the survival index of all *C. striatum* strains, at least at 20 and 22 °C. The effect of atrazine on survival index at 24 °C seemed less pronounced, though there was no statistically significant interaction between temperature and atrazine. The mode of action of atrazine on non-target species has not been extensively tested, but a study on rotifers by Shim et al. [84] showed atrazine to increase antioxidant activity at similar concentrations as used in our study. Since temperature usually increases metabolic activities, the 24 °C treatment might have ameliorated the effects of atrazine-related oxidative stress sufficiently for individuals of *C. striatum* to stay and grow rather than to try and disperse. However, there is also the possibility that a temperature related increase in metabolic oxygen demand combined with increased antioxidant activities exhausted the nergy budget, leaving individuals unable to disperse [39].

Our study supports existing theory and observations suggesting that intraspecific trait variation (ITV) can be equally important for the stability of ecosystem functions in the face of anthropogenically induced environmental change as interspecific trait variation, through its effects on demography [16, 17, 25–31]. Both species tested here differed intraspecifically in their base demography (meaning at control conditions) and strains of *T. thermophila* also differed in their demographic response to the environmental change drivers tested. Regarding survival strategy, we were able to demonstrate that both species shifted towards a flee strategy with increasing intrinsic growth and self-limitation, and that the scale of this shift varied among strains of both species. Therefore, the response to environmental change of a community comprised of these two species would likely depend on the strains contributing to the population of each species, as well as on the interactions among those strains within each species. We did not quantify the interaction coefficients among strains, but doing so for strain combinations within and between species would be an important next step in gaining more empirical evidence and understanding of if and how ITV can modulate environmental change effects on population and, ultimately, ecosystem dynamics.

Population dynamics (in the form of demographic traits) are influenced by the cumulative amount of energy allocated to reproduction. It stands to reason that a change in demography comes with a change in energy allocation, either from reproduction to growth and/or survival strategies or vice versa [85–88]. In this study, we chose our traits of interest based on previous work on *T. thermophila* [72, 89] and we compiled our strategy index in the simplest way possible by letting each trait have equal weight. However, our traits of interest and changes therein probably differ in their energy demands as well as in their importance for the survival strategy expressed by the cell in question. It would be interesting for future work to dive deeper into the specifics of phenotypical trait values, and changes therein, related to specific life strategies in different species of ciliate and other zooplankton grazers.

## Conclusions

Our study provides empirical evidence for a link between population-level demographic and trait responses to environmental change drivers in two species of ciliate. This is but a first step to better understand the modulating role of ITV in environmental change effects on population dynamics. Still, our results support theoretical studies emphasizing the importance of ITV for population and, ultimately, ecosystem dynamics [18, 36]. As a next step, future work should endeavor to test whether ITV can indeed create intraspecific functional redundancy among groups of phenotypically different individuals, and whether increased functional redundancy can indeed mitigate the effects of environmental change drivers on the population as a whole.

### Supplementary Information

**Additional file 1: Supplementary tables S1, S2 and S3.**

**Additional file 2: Supplementary figures S1, S2, S3, S4, S5 and S6**.

## Data Availability

The datasets generated and analyzed during the current study are available in the Open Science Framework repository, https://osf.io/73qgp/?view_only=194685f4763847eeafd77d3d13c9935e.

## Abbreviations

ITV: Intraspecific trait variation
Colp: *Colpidium* *striatum*
Tetra: *Tetrahymena* *thermophila*
