# Supplementary material for: Intraspecific demographic and trait responses to environmental change drivers are linked in two species of ciliate
Source: BMC Ecol Evol. 2024 Apr 17;24:47. doi: 10.1186/s12862-024-02241-2 (PMC11022343; doi:10.1186/s12862-024-02241-2)
Supplement: Supplementary file 2 — Additional file 2: Supplementary figures S1, S2, S3, S4, S5 and S6. [file 12862_2024_2241_MOESM2_ESM.pptx]

## Slide 1
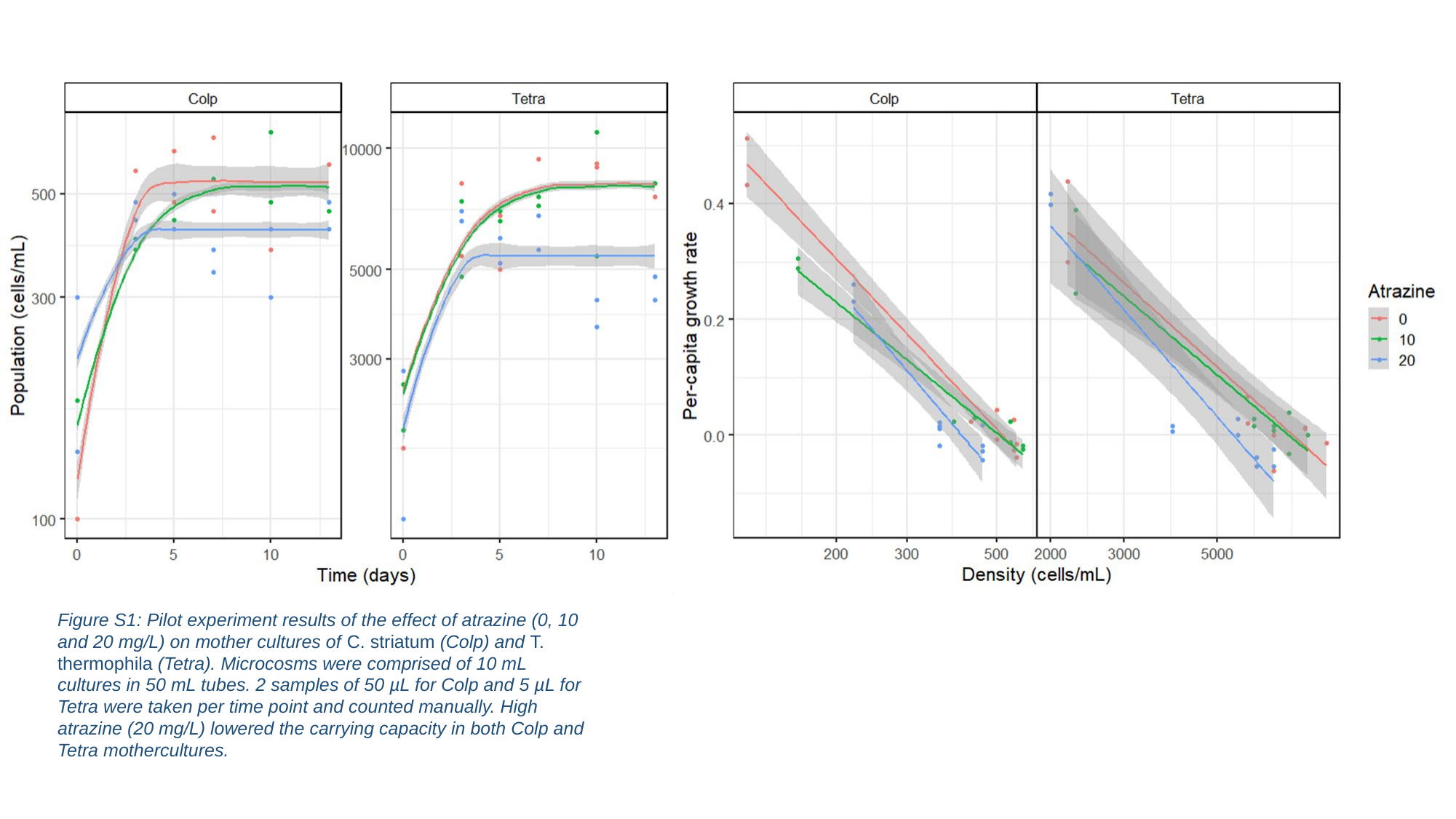

Figure S1: Pilot experiment results of the effect of atrazine (0, 10 and 20 mg/L) on mother cultures of C. striatum (Colp) and T. thermophila (Tetra). Microcosms were comprised of 10 mL cultures in 50 mL tubes. 2 samples of 50 µL for Colp and 5 µL for Tetra were taken per time point and counted manually. High atrazine (20 mg/L) lowered the carrying capacity in both Colp and Tetra mothercultures.

## Slide 2
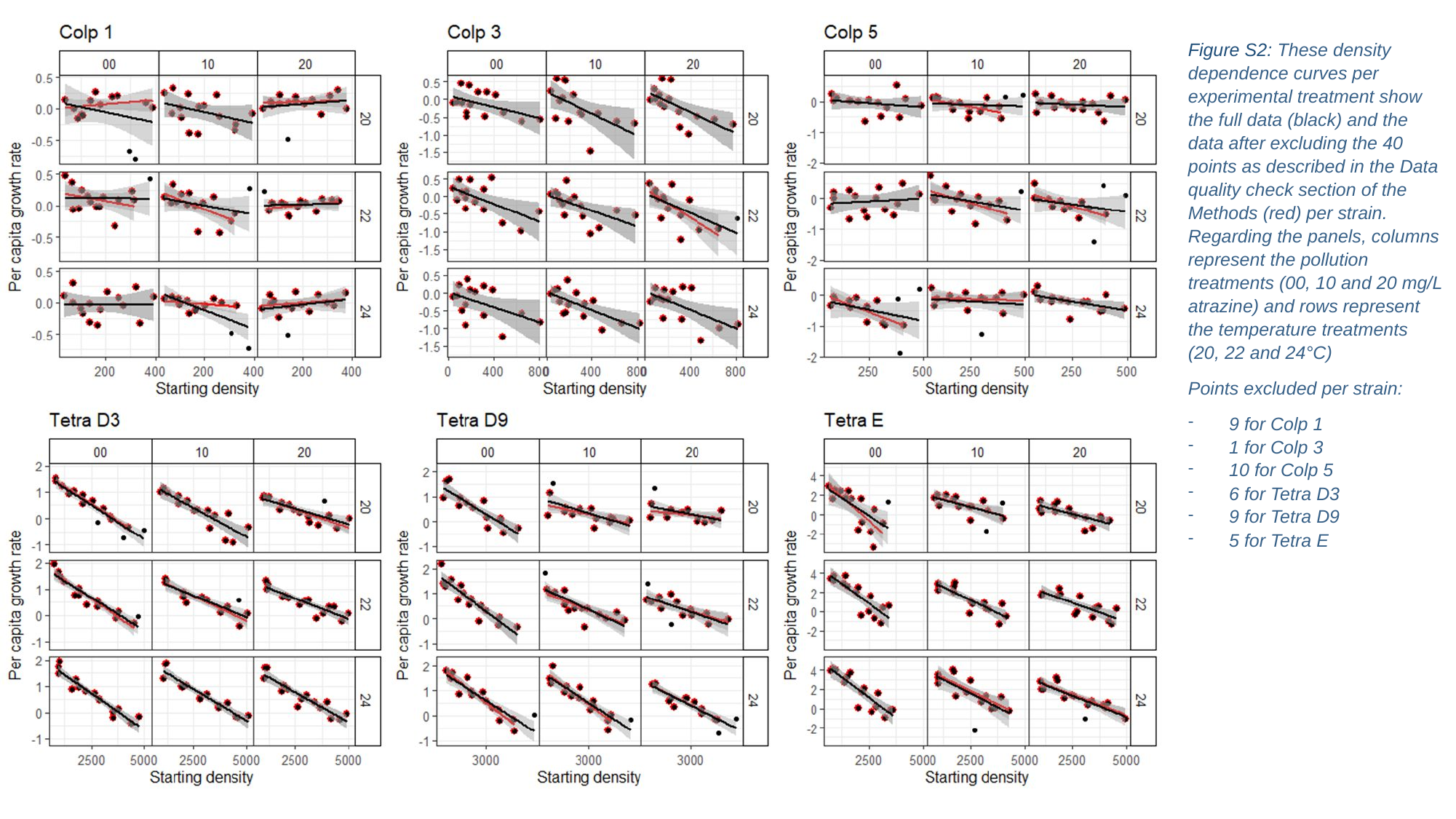

Figure S2: These density dependence curves per experimental treatment show the full data (black) and the data after excluding the 40 points as described in the Data quality check section of the Methods (red) per strain. Regarding the panels, columns represent the pollution treatments (00, 10 and 20 mg/L atrazine) and rows represent the temperature treatments (20, 22 and 24°C)
Points excluded per strain:
9 for Colp 1
1 for Colp 3
10 for Colp 5
6 for Tetra D3
9 for Tetra D9
5 for Tetra E

## Slide 3
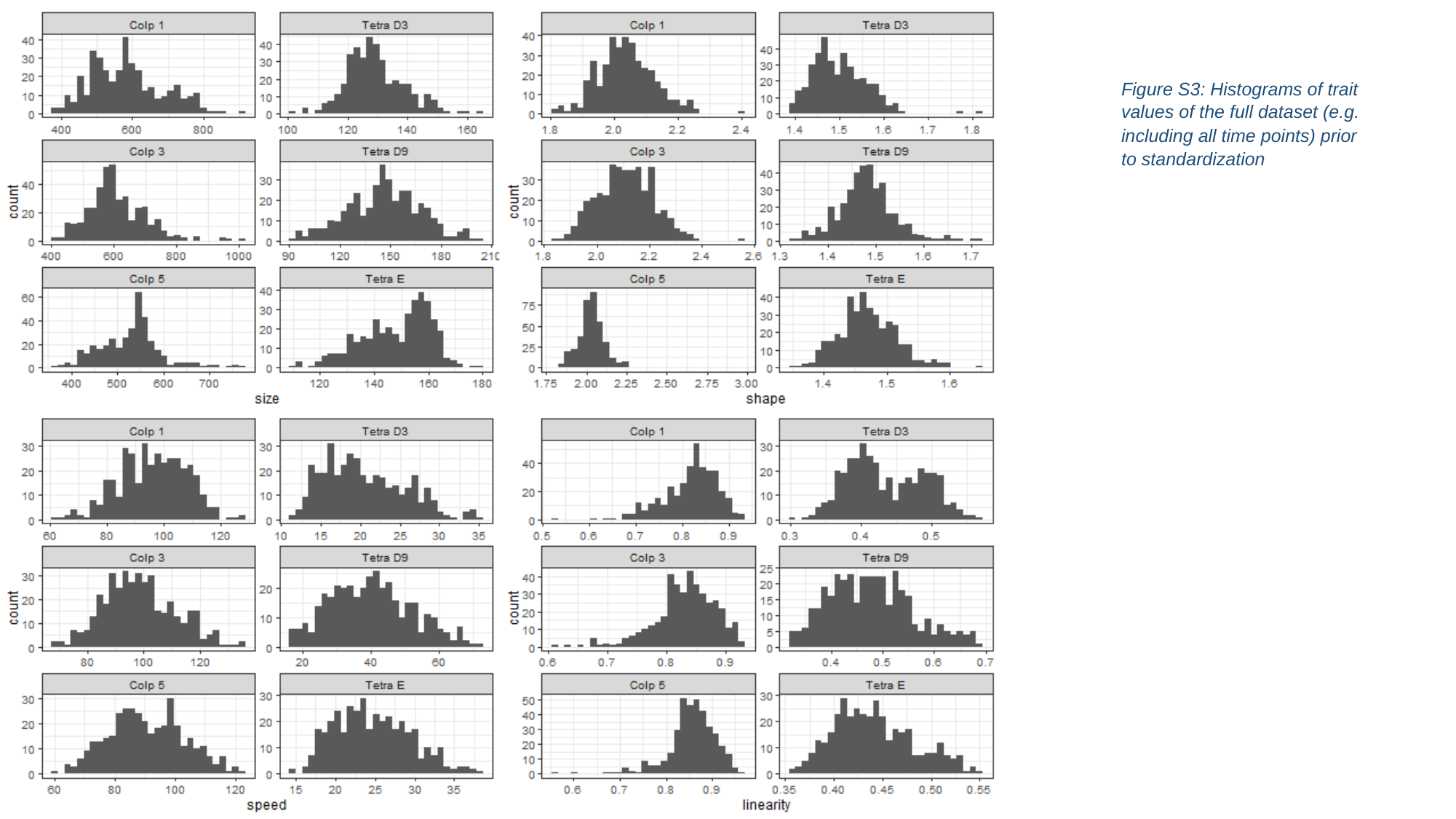

Figure S3: Histograms of trait values of the full dataset (e.g. including all time points) prior to standardization

## Slide 4
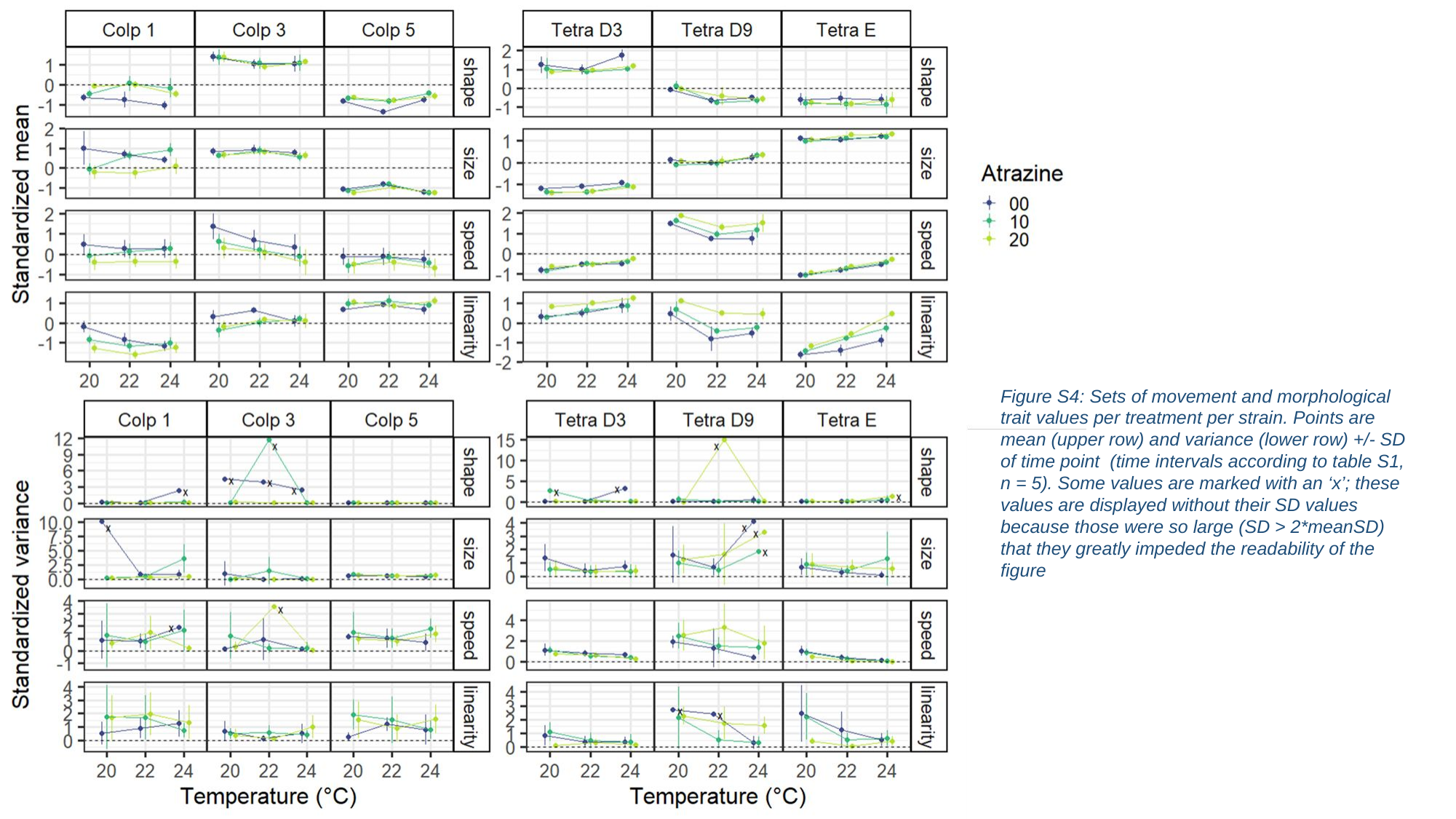

## Slide 5
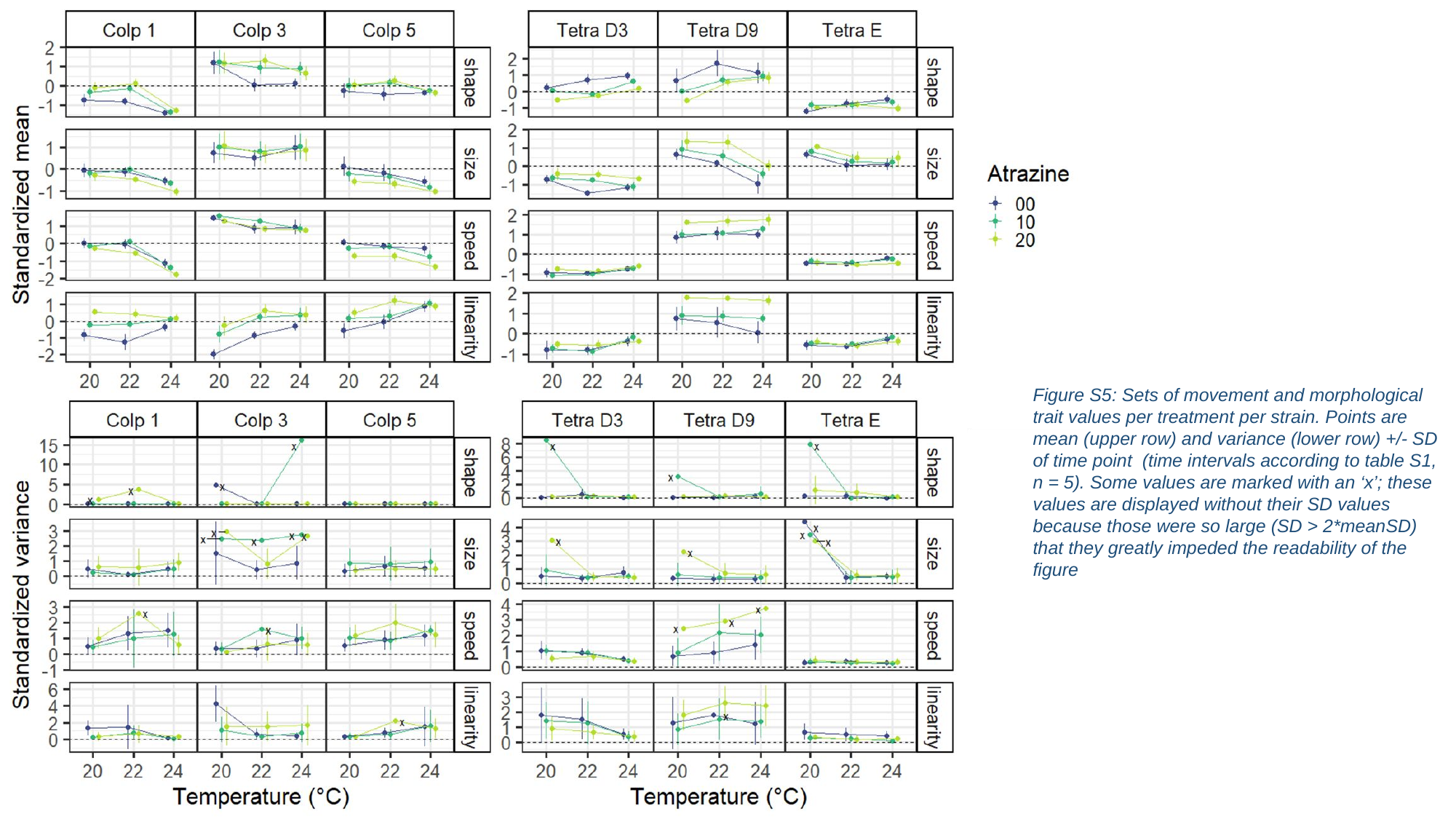

## Slide 6
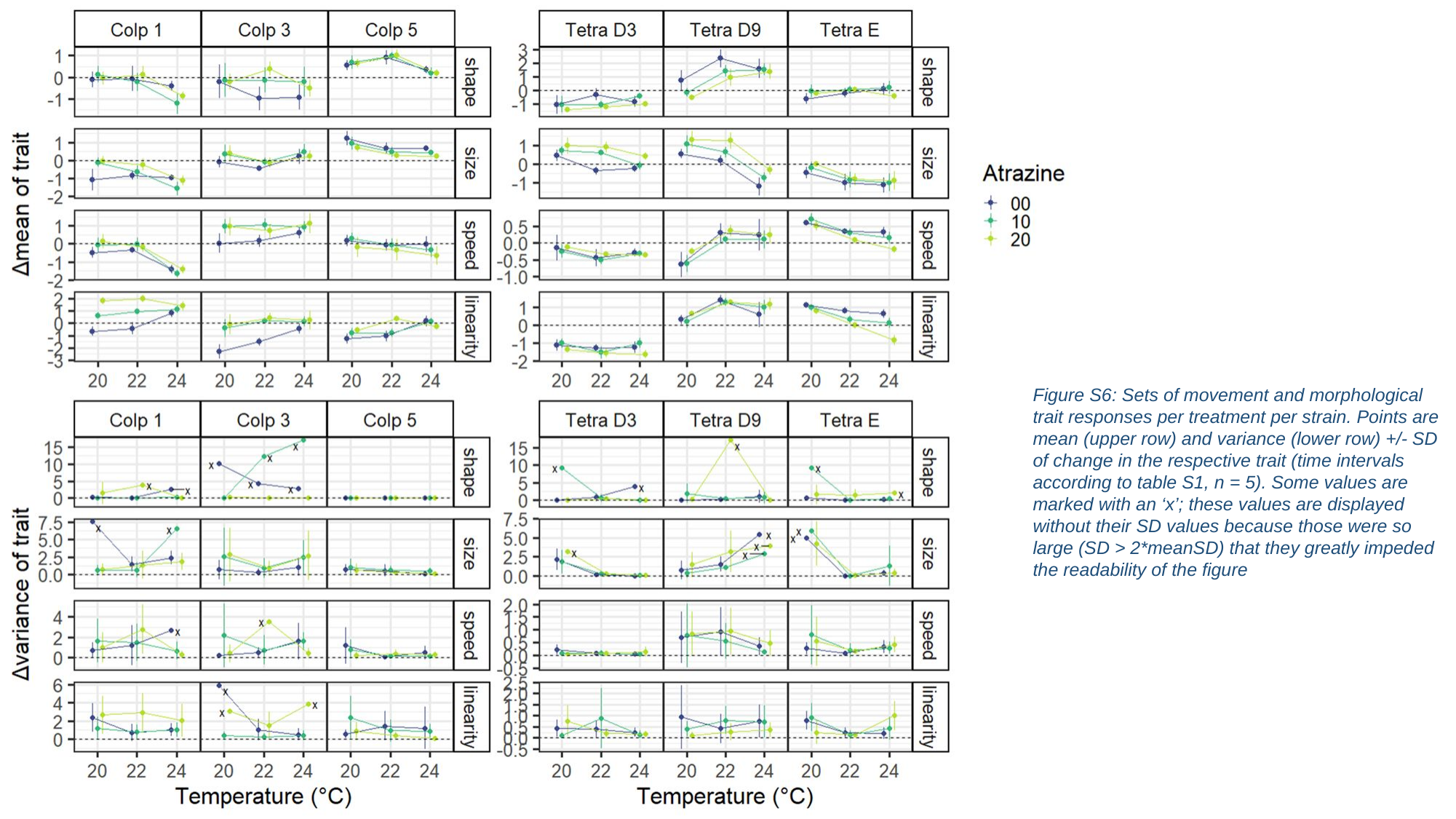

Figure S6: Sets of movement and morphological trait responses per treatment per strain. Points are mean (upper row) and variance (lower row) +/- SD of change in the respective trait (time intervals according to table S1, n = 5). Some values are marked with an ‘x’; these values are displayed without their SD values because those were so large (SD > 2*meanSD) that they greatly impeded the readability of the figure
